# College of Physicians

**Published:** 1841-11-06

**Authors:** 


### PROCEEDINGS OF SOCIETIES.

### COLLEGE OF PHYSICIANS.

At the last session of the College of Physicians of Philadelphia, held Tuesday, November 2d, a committee of three gentlemen was appointed to carry into operation a plan for the publication of a bulletin of their proceedings, which will enable us, hereafter, to comment upon such of their labours as are interesting to the profession at large.

